# Book Reviews

**Published:** 1896-06

**Authors:** 


### Book Reviews.

Twentieth Century Practice. An International Encyclopedia of Modern Medical Science by Leading Authorities of Europe and America. Edited by Thomas L. Stedman, M. D., New York City. In Twenty Volumes. Volume V Diseases of the Skin, New York, William Wood & Co., 1896.

The subjects have been assigned to well-known workers in the province of dermatology who have been selected from special fitness. The chapter on Parasitic diseases has been very properly allotted to L. Duncan Bulkley, who is certainly, by virtue of an experience with 20,000 cases of ringworm, in a position to speak authoritatively. Eczema and dermatitis is discussed by J. Hevins Hyde, of Chicago, in a ninety-page article.

Squamous affections by H. Radcliffe Croeker, of London, one of the most reliable and experienced writers on dermatology.

P.pular Affections, including the vexed question of lichen, are described by L. Brocq, of Paris. Brocq adapts the classification of the Vienna school, of: 1, lichen scrofulosorum. 2, lichen ruber with its varieties, planus alumniatus, obturus, mainliformis, corneus, etc.

Van Harlingen has been given Diseases of the Sebaccous Glands and is well-fitted by reason of his pioneer work on this subject.

The description of Xeroderma figmentorum is given by Prof. Kaposi, of Vienna, who was first to recognize and describe this disease. Benign Neoplamis, John T. Bowen, Boston.

Dermatoneuroses, by Prof. H. Leloir, of Lille, Belgium.

The work reminds one of a benefit performance, in which only well known actors take part, each playing the part which best suits him.

---

Syphilis in the Middle Ages and in Modern Times.—By Dr. F. Buret, Paris, France. Translated from the French, with notes, by A. H. Ohmann, Dumesnil, M. D., Professor of Dermatology and Syphilology in the Marion Sims College of Medicine, etc. Being Volumes II and III of "Syphilis To-day and Among the Ancients," complete in three volumes. 12mo, 300 pages. Philadelphia: The F. A. Davis Co.

This elegant translation of Buret's work finishes the three volumes. It begins with a consideration of syphilis in the middle ages, and brings it down to the present. It is an elaborate and convincing argument against the Columbian origin of syphilis, and it proves by painstaking and elaborate research, that syphilis is not only not of American origin, but is probably a disease of great antiquity. In spite of its paleuric character, the book is extremely interesting, and well worth reading.

---

THE AMERICAN YEAR-BOOK OF MEDICINE AND SURGERY: Being a yearly digest of scientific progress and authoritative opinion in all branches of medicine and surgery, drawn from journals, monographs and text-books, of the leading American and foreign authors and investigators. Under the general editorial charge of George M. Gould, M. D. Profusely illustrated with numerous wood-cuts in text, and 33 handsome half-tone and colored plates. 8vo, pp. 1183. Philadelphia: W. B. Saunders, 1896.

Gould's American Year-Book contains a careful summary and review of the important work done during the year. It is modeled on the English plan of year-books, but is much more comprehensive and full. The contributors are in general well known in the profession, not a few of whom are Atlanta physicians. The volume is of the same size and general appearance as Saunder's American Systems.

---

Manual of the Practice of Medicine. By George Roe Lockwood, M. D. With seventy-five illustrations and seventy-two full page olored plates. W. B. Saunders, Philadelphia, 1896.

In this manual the author has striven to set forth the principles and practice of medicine in a condensed form. The views expressed are entirely in accord with modern opinion, and are abreast with the time. The author believes that the mortality from diphtheria will be greatly reduced by the early use of anti-toxine.

The book is well written, clear and concise enough to be a valuable aid to the student and practitioner.

